# Efficacy of Arytenoidectomy after Suture Lateralisation Failure in Patients with Bilateral Vocal Cord Paralysis

**DOI:** 10.1155/2020/8822164

**Published:** 2020-11-12

**Authors:** Shinsuke Suzuki, Takechiyo Yamada

**Affiliations:** Department of Otorhinolaryngology & Head and Neck Surgery, Akita University Graduate School of Medicine, Akita 010-8543, Japan

## Abstract

**Background:**

Endolaryngeal suture lateralisation is an ideal operation for bilateral vocal fold paralysis. However, restenosis owing to breakage and slippage of suture can sometimes occur. In such a case, methods that are more effective in expanding the glottis, including arytenoidectomy, must be selected. *Case Report*. Herein, we report two female patients aged 86 and 54 years who presented with bilateral vocal cord paralysis and who had restenosis after suture lateralisation. Endoscopic partial arytenoidectomy was performed, and satisfactory outcomes were obtained. This method maintains the height of the arytenoid and preserves its sensation by leaving a part of the cartilage and mucous membrane.

**Conclusion:**

Endoscopic partial arytenoidectomy is effective for securing the airway while preserving vocal function and preventing aspiration. This technique is suitable for patients with restenosis after they have undergone endolaryngeal suture lateralisation.

## 1. Introduction

Bilateral vocal cord paralysis, a condition in which the vocal cords are fixed in the midline, impairs breathing and is considered life-threatening. Several minimally invasive operations are conducted for this condition. However, numerous challenges must be overcome simultaneously, which include securing the respiratory pathways, preserving vocal function, and preventing aspiration.

This procedure can be generally divided into two. The first method is posterior cordectomy or arytenoidectomy, a technique that secures the respiratory tract by excising or removing a part of the structure of the larynx [1, 2]. However, these procedures are irreversible, and wound contracture and aspiration can occur. Therefore, the use of some devices is required to reduce complications.

Another method is endolaryngeal suture lateralisation (ESL) of the vocal fold, which preserves the structure of the larynx [3].

This technique can preserve the structure of the larynx, including the mucous membrane and cartilage, and is widely used with some modifications because it is less invasive and reversible. However, restenosis can sometimes occur owing to breakage and slippage of suture, thereby resulting in loss of suture tension, which can be a serious problem. In these cases, the airway must be secured while preserving vocal function and preventing aspiration.

Herein, we report on two patients with bilateral vocal cord paralysis who had restenosis after undergoing ESL. Endoscopic partial arytenoidectomy (EPA) was performed, and satisfactory outcomes were obtained.

## 2. Case Report

### 2.1. Case 1

An 86-year-old female patient underwent total thyroidectomy for a lymphoid follicular tumour of the thyroid 14 years ago. Postoperative bilateral vocal cord paralysis was observed, and tracheostomy was performed. After 3 years, ESL was performed on the left side, and tracheostomy was closed. Then, the patient did not have any problems with respect to breathing and vocalisation. However, 8 years later, endotracheal intubation was performed while the patient was under general anaesthesia owing to intestinal obstruction. The operation for intestinal obstruction was performed without any complications. However, the patient presented with dyspnoea after extubation, and tracheostomy was performed. Because her respiratory condition did not improved after 1 month, she was referred to our department for treatment.

**Figure 1:**
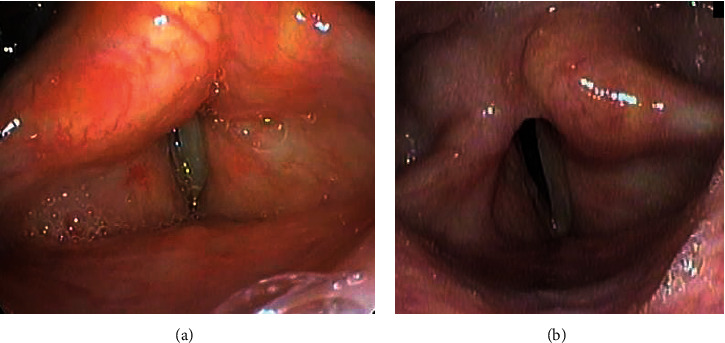
Fiberscopic examination of the larynx in case 1. (a) Pre-endoscopic partial arytenoidectomy (EPA). The bilateral vocal cords were fixed at the paramedian position. Suture pulling of the vocal cords could not be confirmed. (b) Post-EPA. The right arytenoid cartilage has been removed, and the posterior part of the glottis was widened.

Examination of the larynx did not reveal any sutures that would have pulled the left vocal cord, and the bilateral vocal cords were fixed in the midline. Moreover, the glottis was closed (Figure 1(a)).

**Figure 2:**
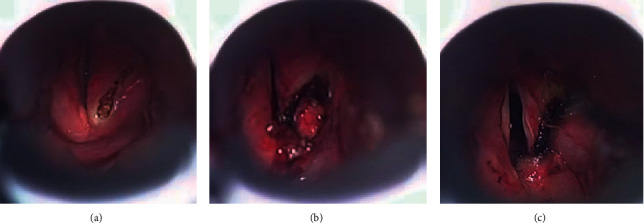
Procedures during endoscopic partial arytenoidectomy. (a) An anterior incision was made with a semiconductor laser in the right arytenoid. (b) The right arytenoid cartilage was visualised and removed partially. (c) The mucous membrane was preserved and fixed with fibrin glue; the glottis was widened.

Transoral partial resection of the right arytenoid cartilage was performed using semiconductor lasers, according to previous reports [4, 5]. The mucous membrane was preserved and fixed with fibrin glue to cover the excised cartilage defect (Figure 2). Three years after the surgery, the patient did not have problems in both vocalisation and swallowing (Figure 1(b)).

### 2.2. Case 2

A 54-year-old female patient underwent subtotal thyroidectomy for thyroid cancer 17 years ago. The patient was diagnosed with postoperative bilateral vocal cord paralysis, and tracheostomy was then performed. The following year, the first ESL was performed on the left vocal cord. The patient's postoperative respiration was good, and the tracheostomy was closed. However, about 1 year later, the patient presented with dyspnoea; thus, tracheostomy was performed again. After the vocal cords were assessed, breakage of the pulling thread was confirmed. Therefore, a second ESL was conducted on the left side. Her respiratory condition improved, and the tracheostomy was closed. However, after 4 years, the patient presented with dyspnoea again; thus, tracheostomy was conducted. A thorough assessment of the vocal cords revealed that the left vocal cords that were towed were relaxed, and the glottis was narrowed. Then, a third ESL was performed on the right side. The patient's breathing improved, and the tracheostomy was closed. However, after 1 month, the patient again presented with dyspnoea. The vocal cords on the right side were loosened, and the glottis was narrowed. Tracheostomy was then performed, and the patient was referred to our department for treatment.

**Figure 3:**
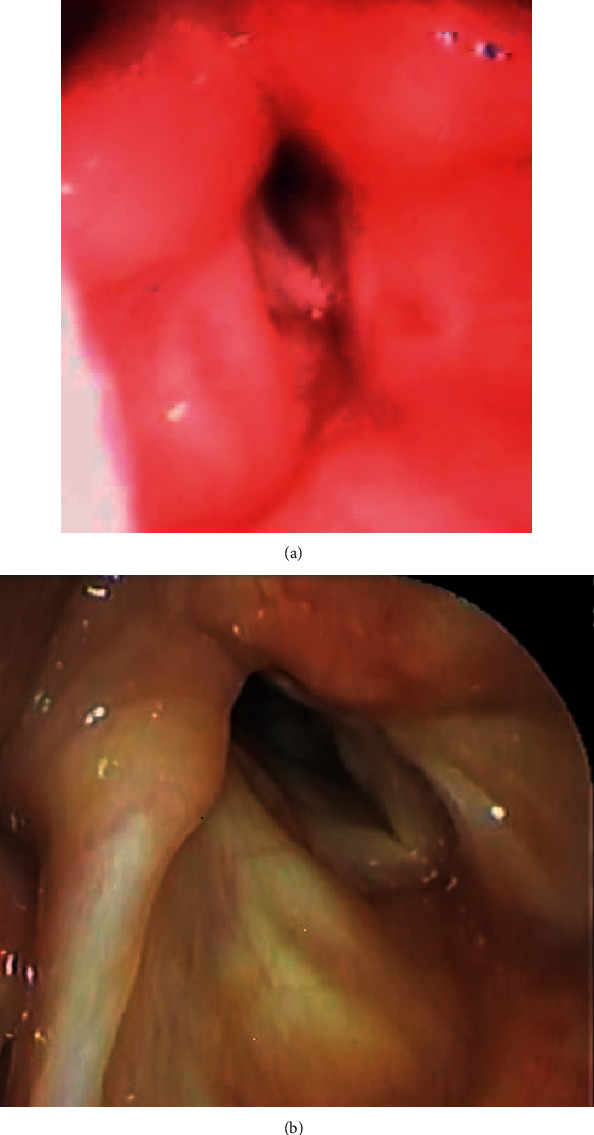
Fiberscopic examination of the larynx in case 2. (a) Pre-endoscopic partial arytenoidectomy (EPA). The right vocal cord, which should have been pulled laterally, was loose and fixed at the paramedian position. (b) Post-EPA. The right arytenoid cartilage was removed, and the posterior part of the glottis was widened.

The left vocal cord was towed laterally but was observed to be poor. The right vocal cords were fixed midway, with no traction (Figure 3(a)).

Transoral submucosal partial resection of the right arytenoid cartilage was performed in our department.

Nine years after the operation, the patient has been living daily without any problems in both vocalisation and swallowing (Figure 3(b)).

## 3. Discussion

Treatment for bilateral vocal cord palsy requires preserving breathing, swallowing, and speech function, and a minimally invasive and highly effective surgery is important.

Several surgical techniques for glottis widening to re-establish an adequate laryngeal airway and acceptable phonation and swallowing have been reported [6]. However, most of these procedures are irreversible and often result in problems, such as scarring or granuloma formation, which causes renarrowing of the airway.

To solve these problems, various solutions have been proposed, which include performing ESL. Kircher first presented the concept of this technique [7], and Ejnell and Tisell have developed the method [3]. This surgery is relatively simple and minimally invasive. In addition, since the procedure is reversible, it is considered advantageous as the traction can be released even when the vocal cord movement recovers in the future. By contrast, the occurrence of restenosis due to tearing or loosening of the thread, which is considered a major problem, has been reported [8, 9]. Different methods that prevent suture problems have been assessed [10].

The cause of restenosis is not only the problem of pulling thread but also the presence of endolaryngeal soft tissue because tension in the muscle is weaker than that in the vocal cords, and the phenomenon that the vocal cords deviate to the inside of the larynx is also considered. In relation this reason, suture lateralisation of the ventricular fold along with the vocal cords has been proposed [8]. However, restenosis cannot be completely prevented with such method.

Herein, we report two cases of restenosis after ESL surgery. In the first case, dyspnoea occurred after the administration of general anaesthesia via endotracheal intubation. Temporary laryngeal oedema due to intubation caused the airway to narrow. Moreover, airway stenosis did not improve after 8 months, and restenosis was considered due to the anaesthesia procedure, including endotracheal intubation. The suture was assumed to be broken, and the fact that no thread was found during EPA supports this notion.

In case 2, the patient previously underwent three ESL procedures. Moreover, a breakage of the suture was confirmed during the first surgery. However, there was no evidence of suture breakage during the second and third operations. However, the soft tissue was loose, and the glottis was narrowed.

The cause of airway stenosis was the collapse of soft tissue due to an increase in pressure inside the larynx caused by the Bernoulli effect [11]. Such phenomenon might have caused the condition. In this case, a high pressure inside the larynx might has resulted in the condition. However, such notion is difficult to confirm.

Thus, a method that can more effectively enlarge the glottis should be selected.

Arytenoidectomy is an irreversible procedure. However, it can effectively enlarge the glottis. In recent years, the efficacy of lasers in such procedures has been reported, and such devices have been widely used [4, 12]. However, if the mucous membrane is burned, the sensation in this part is lost, thereby causing aspiration. In addition, if the cartilage is completely removed, the height of the arytenoid is lost, which can also cause aspiration. In relation to these reasons, partial resection of the arytenoid cartilage is considered effective and safe as it preserves the mucosa [2, 13]. This method maintains the height of the arytenoid and preserves its sensation by leaving part of the cartilage and mucous membrane. Moreover, it can secure an adequate airway while preserving acceptable phonation and not causing an increased risk of aspiration [5].

Although EPA is an irreversible and invasive procedure compared with ESL, it is excellent in preserving vocal function and preventing aspiration. Thus, it is considered suitable for patients with restenosis after ESL.
